# The geometric framework for nutrition reveals interactions between protein and carbohydrate during larval growth in honey bees

**DOI:** 10.1242/bio.022582

**Published:** 2017-04-10

**Authors:** Bryan R. Helm, Garett P. Slater, Arun Rajamohan, George D. Yocum, Kendra J. Greenlee, Julia H. Bowsher

**Affiliations:** 1 Department of Biological Sciences, North Dakota State University, Fargo, ND 58108-6050, USA; 2 Agricultural Research Service – Insect Genetics and Biochemistry, Red River Valley Agricultural Research Center, United States Department of Agriculture, Fargo, ND 58102, USA

**Keywords:** Honey bee, *Apis mellifera*, Nutrition, Geometric framework, Larva, Growth, Diet quality

## Abstract

In holometabolous insects, larval nutrition affects adult body size, a life history trait with a profound influence on performance and fitness. Individual nutritional components of larval diets are often complex and may interact with one another, necessitating the use of a geometric framework for elucidating nutritional effects. In the honey bee, *Apis mellifera*, nurse bees provision food to developing larvae, directly moderating growth rates and caste development. However, the eusocial nature of honey bees makes nutritional studies challenging, because diet components cannot be systematically manipulated in the hive. Using *in vitro* rearing, we investigated the roles and interactions between carbohydrate and protein content on larval survival, growth, and development in *A. mellifera*. We applied a geometric framework to determine how these two nutritional components interact across nine artificial diets. Honey bees successfully completed larval development under a wide range of protein and carbohydrate contents, with the medium protein (∼5%) diet having the highest survival. Protein and carbohydrate both had significant and non-linear effects on growth rate, with the highest growth rates observed on a medium-protein, low-carbohydrate diet. Diet composition did not have a statistically significant effect on development time. These results confirm previous findings that protein and carbohydrate content affect the growth of *A. mellifera* larvae. However, this study identified an interaction between carbohydrate and protein content that indicates a low-protein, high-carb diet has a negative effect on larval growth and survival. These results imply that worker recruitment in the hive would decline under low protein conditions, even when nectar abundance or honey stores are sufficient.

## Abstract (precis)

**Summary:** Protein to carbohydrate ratio affects the survival and growth rate of larval worker honey bees *in vitro*. Protein and carbohydrate content of food interact in complex ways to alter growth rate, development time and survival in worker bound honey bee larvae.

## INTRODUCTION

Nutrition is one of the most important environmental factors that determines both the growth and development of organisms. Dietary nutrients are not only needed for respiration and metabolism to fuel growth and development but also to provide essential chemical building blocks used for tissue construction and overall growth. Thus, nutrition serves as a *sine qua non* for both growth and development and shapes phenotypic variation, such as body size (Chown and Nicolson, 2004; Simpson and Raubenheimer, 2011). Despite its central importance, the roles particular nutritional components play in overall growth and development are not well characterized in many organisms, and non-linear interactions among specific diet components make nutrition difficult to study (Raubenheimer et al., 2009). The geometric framework for nutrition provides a standardized approach for studying effects of nutrition, and a robust conceptual framework for exploring the effects of nutritional complexity on performance and fitness (Behmer, 2009a,b; Harrison et al., 2014; Raubenheimer and Simpson, 1994, 1999; Simpson and Raubenheimer, 1995, 2001, 2011). This framework allows researchers the ability to disentangle the effects of major macronutrients, such as protein and carbohydrate, and robustly captures the complexity of nutritional effects on growth.

In holometabolous insects, larval nutrition plays a critical role in shaping adult phenotypes (Boggs, 2009; O'Brien et al., 2002, 2005). Many aspects of the adult phenotype become fixed at metamorphosis (Boggs, 2009), which can lead to variance in adult morphometrics related to performance and reproduction (Chown and Gaston, 2010; Emlen and Allen, 2003; Shingleton et al., 2009; Stern and Emlen, 1999). Perhaps the most striking examples of nutritionally mediated plasticity occur in eusocial Hymenoptera, which develop into two or more distinct phenotypic castes depending on differences in the quality or quantity of nutritional provisions during juvenile development (Hartfelder et al., 2006; Hrassnigg and Crailsheim, 2005; Libbrecht et al., 2013; Linksvayer et al., 2011; Wheeler et al., 2014). Thus, eusocial Hymenopterans may be especially sensitive to the nutritional composition of larval food sources.

Larval nutrition shapes developmental trajectories in eusocial hymenopterans (Behmer, 2009a) but has proved difficult to study because larval diets are completely controlled by workers. In honey bees, nurse workers use specialized glands to provision larvae with jellies, which they mix with honey and sometimes pollen (Haydak, 1970). These jellies constitute the protein component of larval diets, and also contain carbohydrates, vitamins, sterols and other lipids. Nurse bees modulate the relative proportion of proteins and carbohydrates they provide to larvae depending on larval stage, sex, and caste (Brouwers et al., 1987; Wang et al., 2015). In addition, the nutritional components of jellies change with seasonal shifts in floral resources (Howe et al., 1985; Wongchai and Ratanavalachai, 2002). Because nurse bees mediate larval nutrition, it is methodologically difficult to correlate growth and development in the hive setting to the nutritional composition of jelly fed to individual larva. Studies of individual dietary effects on growth trajectories cannot detect significant interactions between diet components, which could negate effects of individual nutrients. Use of the geometric framework highlights these interactions by manipulating diet components in a methodical way (Raubenheimer et al., 2009), but no studies have used a geometric framework approach to investigate the effects of nutritional components on larval performance in *A. mellifera*.

*In vitro* rearing techniques required to systematically manipulate larval diets in *A. mellifera* have proved challenging and, thus, have not been widely employed. Recent advances in *in vitro* rearing (Aupinel et al., 2005; Crailsheim et al., 2013; Kaftanoglu et al., 2011; Linksvayer et al., 2011) make possible the application of the geometric framework to larval honey bee nutrition. In this study, we use *in vitro* rearing to determine how protein, carbohydrate, and protein×carbohydrate ratios affect survival, growth rate, and development time during the larval stage. Proteins and carbohydrates both are major macronutrients of honey bee diet (Haydak, 1970), affect growth when manipulated separately (Asencot and Lensky, 1977; Brouwers et al., 1987), and have been the focus of other studies using the geometric framework in adult hymenopterans (Dussutour and Simpson, 2012; Paoli et al., 2014; Pirk et al., 2010). We formulated nine diets with different protein and carbohydrate contents using commercial royal jelly and sugars. We reared larvae *in vitro* and analyzed survival, growth rate, and development time using non-linear regressions and performance landscapes. We found that larvae survive and grow on a wide range of nutritional conditions, and there were unexpected interactions among diet components that altered larval growth rates, but not development time.

## RESULTS

### Analysis of royal jelly content and nutrient contents of artificial diets

Protein content of the royal jelly was 12.35%. The non-protein material in the jelly was 27% carbohydrate, and 56% water. Using these values, we calculated the relative protein, carbohydrate, and water contents, as well as the protein to carbohydrate ratios of our different diet treatments (Table 1).

**Table 1.**
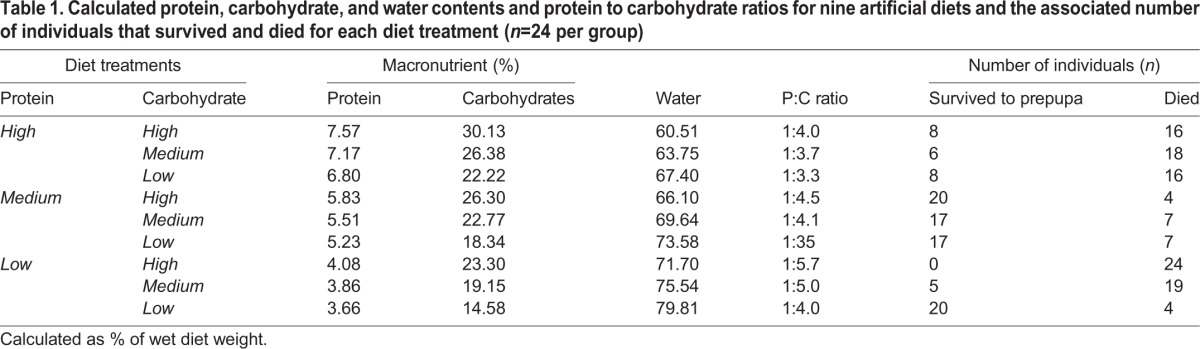
**Calculated protein, carbohydrate, and water contents and protein to carbohydrate ratios for nine artificial diets and the associated number of individuals that survived and died for each diet treatment (*n*=24 per group)**

### Survival on different diet treatments

Protein content, carbohydrate content, and an interaction between protein and carbohydrate contents had significant effects on survival (Table 2). Protein did not have a significant nonlinear effect on survival, because the quadratic term was insignificant in this analysis. However, survival was significantly affected by carbohydrate content in a nonlinear manner, as the quadratic term in our model had a significant effect (Table 2).

**Table 2.**
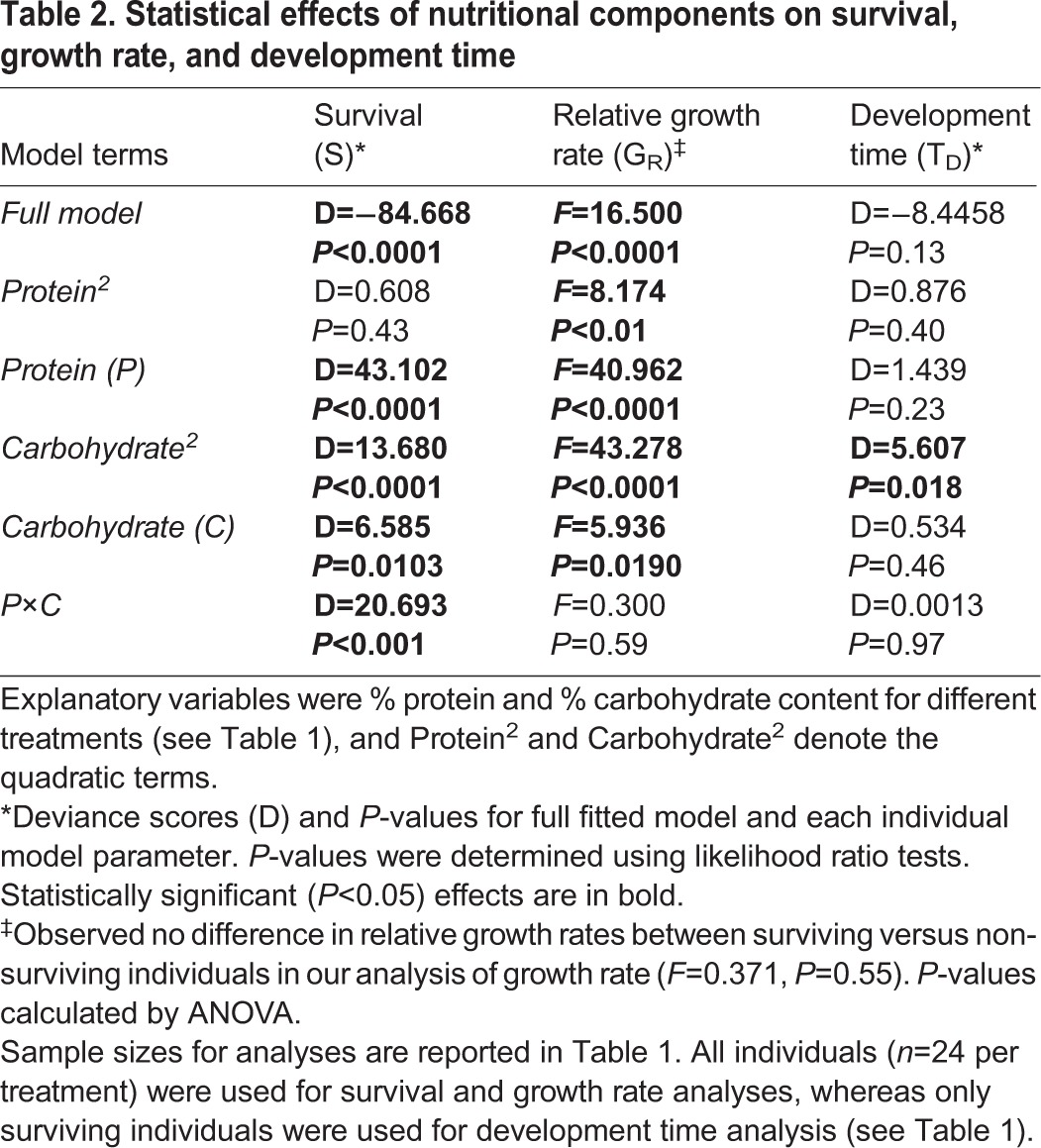
**Statistical effects of nutritional components on survival, growth rate, and development time**

**Fig. 1.**
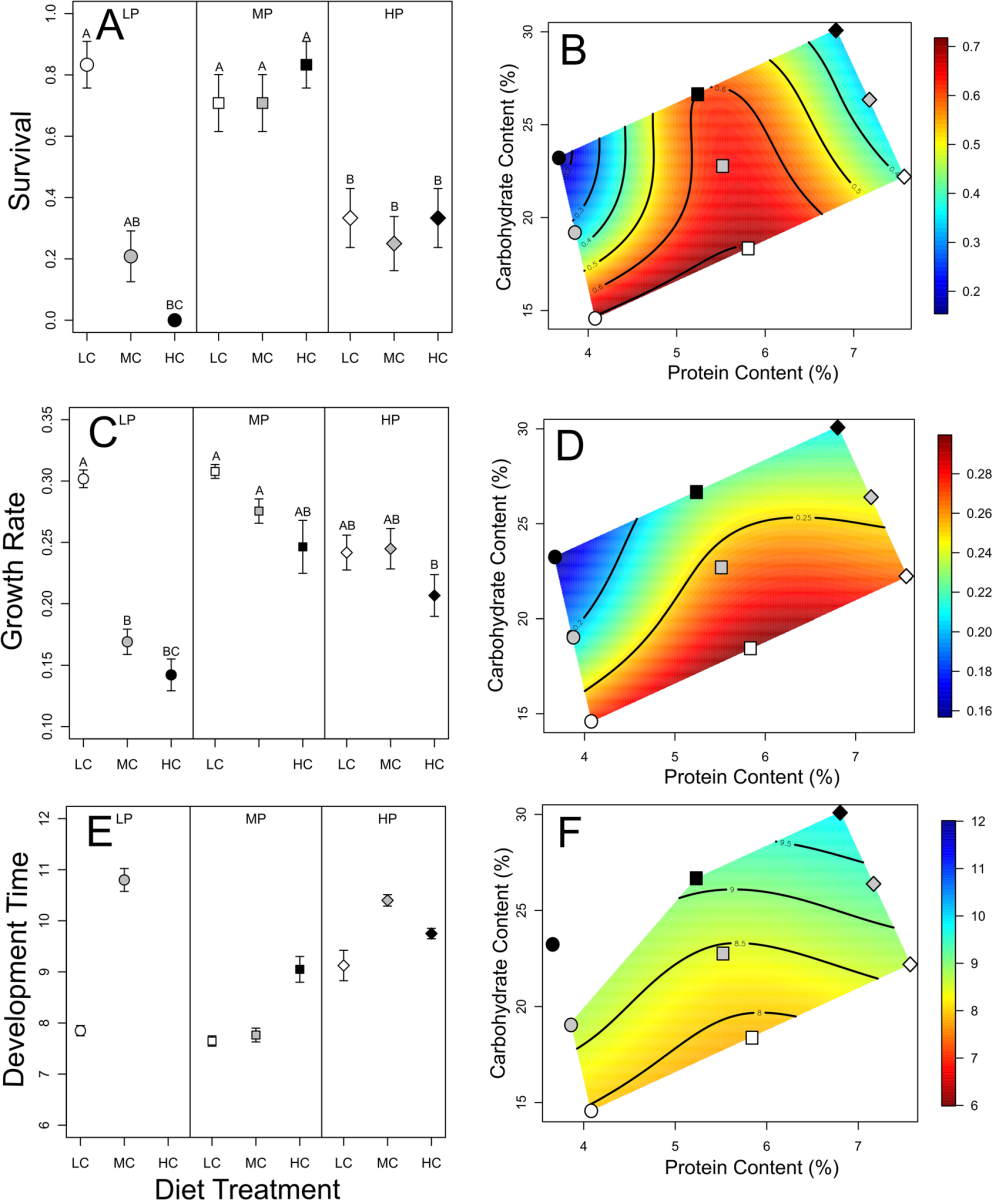
**Larval growth metrics and performance landscapes in response to different protein and carbohydrate contents of food.** Proportion of individuals that survived to the prepupal stage (A,B), mean growth rate *G_R_* (C,D), and development time *T_D_* (E,F) of *A. mellifera* larvae reared on different artificial diet treatments. Low protein (LP, circles), medium protein (MP, squares), and high protein (HP, diamonds) are separated by lines. Low carbohydrate (LC, white), medium carbohydrate (MC, gray), and high carbohydrate (HC, black) combinations are shown for each level of protein treatment. Error bars represent standard error of proportions (A) or standard errors of means (C,E) for each treatment, while letters within graphical panels indicate significant post hoc differences among treatments, see text for descriptions of appropriate post hoc tests (*P*<0.05; pairwise comparison of proportions in A; Tukey's test for honest significant differences in C and E). Survival to the prepupal stage (B), growth rate (D), and development time (F) of *A. mellifera* larvae plotted as topographical performance landscapes with respect to protein and carbohydrate concentrations. Diet treatments used to estimate the landscape topography are represented by symbols. Warm colors indicate high values with respect to each measure, and cool colors indicate low values.

Survival was highest overall in the medium protein treatment for low, medium, and high carbohydrate combinations (Fig. 1A). When the protein content of artificial diet was increased, survival decreased by ∼60% on average across low, medium, and high carbohydrate combinations (Fig. 1A). However, survival varied with respect to different carbohydrate treatments when protein was reduced. Under low protein conditions, survival remained high in combination with the low carbohydrate treatment, but declined to 0 as the added carbohydrate content increased (Fig. 1A). These patterns were further supported when survival was mapped onto a performance landscape for protein and carbohydrate concentrations (Fig. 1B). Mortality at high protein concentrations was independent of carbohydrate concentration (Fig. 1B).

### Growth rate on different diet treatments

Protein and carbohydrate contents had significant linear and nonlinear effects on relative growth rates among the different diets (Table 2). We observed no significant differences in relative growth rate between individuals that survived until the prepupal phase and those that died during larval growth (*F*=0.371, *P*=0.55), which justified combining both surviving and non-surviving individuals in this analysis. We also observed no significant interaction between protein and carbohydrate contents (Table 2), indicating that protein and carbohydrate have independent, significant effects on observed growth rates.

Comparing among treatments (Fig. 1C), relative growth rate was highest in the medium protein treatment (versus low: Tukey HSD, Δ=0.078, 95% CI=0.05-0.11, *P*=<0.0001; versus high: Tukey HSD, Δ=0.046, 95% CI=0.019-0.074, *P*<0.001). The second highest growth rate was in the high protein treatment (Tukey HSD, Δ=0.032, 95% CI=0.0040-0.060, *P*=0.024). Across all protein treatments, less carbohydrate in the artificial diet resulted in significantly higher growth rates than medium (Tukey HSD, Δ=0.052, 95% CI=0.024-0.080, *P*<0.0001) and high (Tukey HSD, Δ=0.082, 95% CI=0.055-0.110, *P*<0.0001) carbohydrate treatments. Medium carbohydrate treatments resulted in greater relative growth rates than high carbohydrate treatments (Tukey HSD, Δ=0.0303, 95% CI=0.0026-0.0579, *P*=0.029). While an overall interaction term was not significant, the effect that carbohydrate levels have on relative growth rate was more pronounced when protein content is low (Fig. 1C).

Mapping growth rates onto a performance landscape between varying protein and carbohydrate levels, intermediate protein content along with low carbohydrate content showed the highest relative growth rates (Fig. 1D). Increasing or decreasing protein contents resulted in net reductions in relative growth rate, as did increased carbohydrate content of diet (Fig. 1E). For both high and low protein concentrations, low carbohydrate treatments had faster growth rates than high concentrations of carbohydrates (Fig. 1D).

### Development time on different diet treatments

Artificial diet treatment did not affect development time. The statistical model did not explain variance of development times among the treatments (Table 2: Analysis of Deviance, D=−8.458, *P*=0.13), and terms describing protein and carbohydrate contents were not significant with the exception of the quadratic term for carbohydrate (Table 2). Graphically apparent mean differences (Fig. 1E) were not supported by statistical analysis. The low protein×high carbohydrate treatment had 100% mortality (Table 1, Fig. 1A), which prevented estimation of development time. In summary, protein and carbohydrate have little effect on development time among different diets and resulted in an overall flat topography in the performance landscape (Fig. 1F).

## DISCUSSION

### The effects of nutrition on larval survival

The results demonstrate that both protein and carbohydrate concentrations have strong effects on the proportion of individuals that survive the larval growth period. At intermediate protein concentrations, larval survival is quite high, with approximately 80% of the individuals reaching the prepupal stage of development (Fig. 1A,D), a rate comparable with other studies using *in vitro* techniques for rearing larval *A. mellifera* (Kaftanoglu et al., 2011; Linksvayer et al., 2011). As we raised protein concentrations in larval provisions, there were overall net reductions in larval survival (Fig. 1D). While better developmental performance might be expected if protein concentrations were increased (Chown and Nicolson, 2004), adult honey bees and other hymenopterans show mortality under high protein diets (Dussutour and Simpson, 2012; Pirk et al., 2010).

Interestingly, when protein concentrations were reduced, larval survival became dependent on the concentration of carbohydrates (Fig. 1D). Survival was high – even comparable with our medium protein treatments – when larvae were reared on the low protein×low carbohydrate artificial diet treatment. However, mortality increased substantially as carbohydrate concentrations increased at low protein concentrations. This effect grew more pronounced in the low protein×high carbohydrate treatment, such that 100% of the individuals in this treatment died before completion of larval growth. This may explain the significant non-linear effect of carbohydrates observed in the statistical analysis of development time (Table 2).

### Effects of nutrition on growth rate

The results show that protein and carbohydrate concentrations play an important role in determining the growth rate of larval *A. mellifera*. As with this study of larval survival, larval growth rates were significantly impacted by protein and carbohydrates in complex and non-linear ways. However, unlike survival, growth rates were affected independently by carbohydrate and protein concentrations, because no significant statistical evidence was detected for their interaction (Table 2). Generally speaking, intermediate protein treatments showed the fastest growth with reductions in relative growth rates as protein concentrations were increased or reduced. With respect to carbohydrates, the highest growth rates were consistently associated with low carbohydrate treatments while the lowest growth rates were observed in high carbohydrate treatments. Thus, we suspect that relative growth rates are highest when the protein to carbohydrate ratio is low.

### Effects of nutrition on development time

In contrast to the strong effects of nutrition on larval survival and growth rates, development times were unaffected by the relative nutrient composition of the larval provisions. Despite strong reductions in overall nutrient composition and severe changes in the relative balance between those nutrients, we observed no differences in development time among our artificial diet treatments. The simplest explanation for this pattern could be insufficient sample size due to high mortality. Unlike other growth metrics, only surviving individuals have a development time. In some cases entire treatments died before completion of larval growth (Fig. 1C) – reducing the power to discern biologically relevant differences among treatments.

It is also possible that development time is canalized with respect to nutrient quality in larval provisions. Multiple lines of evidence suggest that development time does not vary within honey bee castes. In combination with a high degree of growth rate variation (Rembold et al., 1980, and this study), honey bee colonies would be expected to have wide variation in adult body sizes – at least within a caste, which are presumably fed relatively similar quantities of food (Linksvayer et al., 2011). Gross differences in size among castes could potentially arise as either differences in growth rate or the duration of growth (Davidowitz and Helm, 2015; Nijhout et al., 2014; Shingleton, 2011) – each responding to differences in provision quality and quantity separately. This is precisely what has been observed in related bumble bees (Couvillon et al., 2010; Hartfelder et al., 2006), which vary in size and caste as a consequence of food quantity rather than quality per se. Variance in nurse attentiveness (Couvillon and Dornhaus, 2009), tradeoffs in worker quality/cost (Couvillon et al., 2010, 2011), or even ecological variability could lead to differences in the nutritional aliquots provisioned by workers, which in turn lead to variation in body size. Other studies have noted that provisioning honey bee larvae *in vitro* with different quantities of food changes caste of the developing larvae (Linksvayer et al., 2011), which differ in their development times and body size (Rembold et al., 1980). Varying quantities and qualities of food may have independent effects on larval growth metrics – growth duration versus growth rates. Our study kept the quantity of food constant (160 µl) across diet treatments, and therefore we would not predict a difference in development time.

### Implications for honey bee nutrition

The geometric framework reveals interactions between diet components and how their balance affects growth and survival. In our study, an unbalanced protein to carbohydrate ratio may explain why high protein concentrations were associated with high mortalities. The high protein diets did not provide enough carbohydrates to balance the protein intake. A potential hypothesis is that mortality may be dependent on the quantity of protein and relative concentration of carbohydrate rather than the absolute effects of either (Table 1). Our commercial source of royal jelly contained too much protein to be used directly for *A. mellifera* larvae – a P:C ratio of 1:2 when 1:4 is optimal based on our results. In support of this, larvae reared *in vitro* on commercial royal jelly as the only diet component have very high mortality rates (Kaftanoglu et al., 2011, and our data not shown). Commercially available jellies have different compositions than jellies provided to larvae due to adulteration during the extraction process (Howe et al., 1985). Though we attempted to balance our experimental effects, the complex chemical composition of royal jelly led to different P:C ratios in our artificial diet provision treatments, and mortality was strongest when this ratio was high. However, high P:C ratios in the medium protein treatment did not result in the severe reductions of larval survival that occurred under low protein treatments (Table 1). Taken together, these results suggest that mortality has a complex relationship with nutrition, especially protein and carbohydrate balance.

Many studies have analyzed the composition of royal jelly, while only a few have cross-examined worker and royal jelly (Haydak, 1943; Wang et al., 2015). Wang et al. (2015) showed that protein is higher in royal jelly than worker jelly and protein content declines in both jellies at a similar rate throughout development. However, an earlier study found that the protein content in worker jelly is higher than that in royal jelly, and the protein content of worker jelly decreases over time, whereas royal jelly protein content increases with time (Haydak, 1943). Perhaps increased protein components are derived from the observation that worker larvae receive some pollen mixed into their jelly provisions, whereas queens do not (Haydak, 1970). Our results indicate that increasing protein will result in decreased growth rates, which would be more likely to result in production of worker caste. However, the protein composition of different jellies may not be important during the beginning stages of caste determination because queens and workers receive similar protein-laden food (Haydak, 1970) and have similar growth rates (Stabe, 1930).

In contrast to protein, carbohydrate content consistently differs between royal and worker jellies. Wang et al. (2015) and Asencot and Lensky (1985) found that royal jelly had significantly more carbohydrate until day 5 when the carbohydrate content increased in worker jelly due to honey incorporation (Haydak, 1970). Day 5 is after the queen and worker have already diverged developmentally (Asencot and Lensky, 1985). Although Kaftanoglu et al. (2011) showed more queens and intercastes were obtained when carbohydrate content was high in their artificial diet, we observed lower growth rates when carbohydrate content was high across all three protein treatments. Wang et al. (2015) observed that the P:C ratio increased during development for the worker, whereas the ratio stays relatively the same for queens.

One major difference between our study and much of the previous work in larval *A. mellifera* nutrition is that the effects of dietary components were experimentally manipulated in isolation from hive dynamics. Furthermore, we reduced the potential confounding factor of caste differences by choosing a rearing technique that results exclusively in workers (Aupinel et al., 2005). Thus, a portion of the described variability in developmental metrics among previous studies in *A. mellifera* (Rembold et al., 1980) may be attributed to the complex effects of macronutrients in nurse-provisioned larval diets. Although there are upper and lower limits required for viability, our results demonstrate that the nutritional investment of developing individuals may vary substantially while still resulting in the production of surviving larvae. This robustness suggests that colonies can successfully rear larvae in a variety of ecological contexts, especially because nurse bees modulate the nutritional environment of larvae through provisioning.

Adult nutrition is expected to differ from larval nutritional requirements because larvae need more protein for growth and development (Haydak, 1970). The geometric framework has been applied to adult honey bees (Altaye et al., 2010; Paoli et al., 2014; Pirk et al., 2010), and supports the general conclusion that adult honey bee workers have lower protein requirements than larvae, as measured in our study. Workers can subsist on carbohydrates alone (Pirk et al., 2010), but prefer some protein early in adult life while shifting to diets higher in carbohydrates as they transition to foraging (Paoli et al., 2014). The range of protein to carbohydrate ratios that support survival in adult workers is much wider than the range required for survival of larval workers in this study. However, the intake ratios for nurse bees may align more closely with larval requirements because nurse bees produce jelly for larvae. Target intake ratios for protein and carbohydrates vary widely between studies, and young adult workers prefer intake ratios from 1:11 (Altaye et al., 2010) to 1:50 (Paoli et al., 2014). When young adult workers are kept with a queen, the protein demand increases, and the optimal P:C ratio for survival becomes 1:3 (Pirk et al., 2010). This increase in protein demand may reflect an expectation that brood will be present, and therefore more protein will be required for jelly production compared to when the queen is absent and no possibility of brood exists. The optimal 1:3 ratio of nurse bees (Pirk et al., 2010) is similar to the 1:4 ratio we observed as optimal for worker larvae, reinforcing the idea that nurse bee protein requirements will be similar to larval requirements because of jelly production.

Studies of geometric patterns for nutrition frequently examine organisms that are capable of foraging between different sources of food to obtain developmental optima. This is certainly not the case for larval *A. mellifera*, which feed on provisions supplied by nurse bees in the colony. Nurse bees acquire macronutrients to synthesize jellies from honey, pollen, and bee bread that are stored in the hive (Haydak, 1970). Larval jelly provisions are biosynthesized by nurse workers such that larval provisions might be compositionally consistent. However, this is not supported when surveying the literature because jelly compositions depend on season, colony level nutritional substrates, and geographic location (Karaali et al., 1988; Wongchai and Ratanavalachai, 2002; Sabatini et al., 2009; Daniele and Casiabianca, 2012). *A. mellifera* larvae are not capable of foraging optimally, but rather are limited by the provisions supplied by workers, which may in turn be at the whim of colony-level nutrition (Behmer, 2009a). Alternatively, workers may not be capable of assessing larval optima and provision larvae with available resources regardless of P:C ratios, necessitating larval robustness to nutritional variation.

A limitation of our inference about the effects of larval nutrition is that completely artificial diets for larval *A. mellifera* have not been developed, and protein cannot be entirely disentangled from the effects of other royal jelly macronutrients using this specific experimental approach. Recent advancements for high-volume, *in vitro* rearing still require the use of commercially harvested honey bee royal jelly in artificial diets (Aupinel et al., 2005; Kaftanoglu et al., 2011). Because the nutritional composition of royal jelly can be variable and include many dietary elements beyond proteins and carbohydrates, this contributes to the difficulty of producing a standardized artificial diet. The effects we observed are potentially an effect of these confounding dietary factors, such as lipids and trace elements, rather than protein alone. Likewise, the effects of protein were not completely isolated by our experimental approach, and effects of increased or decreased royal jelly components may mask the true effect of protein in particular. Royal jelly contains dietary components that have been shown to alter growth and development – especially with regards to caste, such as royal actin, 10-HDA, or pantothenic acid (Kawamura et al., 1999; Wang et al., 2015). These compounds also occur in worker jelly but at different concentrations (Wang et al., 2015). Although they have become associated with caste determination, these compounds also form the basis of the effects observed at the macronutrient scale (Brouwers et al., 1987), and each compound is classified as a protein, carbohydrate, or lipid. An important question to resolve for understanding *A. mellifera* nutrition is how to conceptually and methodologically separate the distinct effects of individual chemical constituents in provisions from their gross effects as dietary macronutrients and vice versa.

An additional limitation of *in vitro* honey bee rearing is that we were unable to ensure survival through pupation for this study. Some individuals survived to adulthood, but relatively few (data not shown). High mortality in the prepupa stage has been observed in previous studies that employed *in vitro* rearing (Kaftanoglu et al., 2011). After larva finish eating, prepupa must be transferred to new containers to successfully complete development (Aupinel et al., 2005; Kaftanoglu et al., 2011). This necessary handling of sensitive prepupa was the likely cause of mortality in our study. Our subsequent experiments have improved prepupal survival by adjusting handling methods (B.R.H, G.P.S., A.R., G.D.Y., K.J.G., J.H.B., unpublished data). Because of high prepupal mortality, we limited the scope of our results to developmental metrics of the larval stage alone. We measured the end of the growth phase by larval defecation, which starts the prepupal stage. Up to that point, our larval survival was comparable to what had been found under similar diets (Aupinel et al., 2005) to the extent that similar diets had been tested. However, our high prepupal mortality means that we cannot make inferences about survival to adulthood on these diets, a limitation that would cause us to overestimate survival. Some diets might support development to the prepupal stage, but are not sufficient to allow metamorphosis or adult eclosion. The technique of *in vitro* rearing as a whole has not yet progressed to a state where larvae can be reliably reared on artificial diets and in artificial environments, pupate, and successfully eclose as adults. Nevertheless, the geometric framework for nutrition still serves as a powerful tool for drawing connections between nutrition and larval development, and future studies could build upon this current study by addressing these limitations.

Our results demonstrate that interactions between protein, carbohydrate, and their ratio in a diet are important for survival of larval honey bees. The decision to test protein and carbohydrate components was motivated by the fact that both are major macronutrients of diet (Haydak, 1970), both have roles in caste-related growth differences (Asencot and Lensky, 1977; Brouwers et al., 1987), and they are the primary macronutrients manipulated in other studies using the geometric framework in hymenopterans (Dussutour and Simpson, 2012; Paoli et al., 2014; Pirk et al., 2010). This study brings established nutritional manipulation methods into the honey bee model system, in which nutrition is a pressing concern due to extensive colony losses (Di Pasquale et al., 2013; Smart et al., 2016). Future studies can build upon the foundation established by this experiment to test the role of other macro- and micronutrients, when the ability to systematically manipulate these components in artificial diets becomes available.

### Conclusions

By applying the geometric framework for nutrition to the problem of honey bee larval development, our study advances the understanding of larval nutrition in *A. mellifera*. Here we use the framework to characterize the effects of nutrition on larval development. Our results demonstrate that successful development to the pupal stage can occur over a wide range of diets, but that growth and survival of larva are influenced by protein and carbohydrate content. In the case of survival, the interaction between these two factors is also important. Future studies of larval nutrition in *A. mellifera* could use this approach to examine the effects of other nutritional components on even more metrics of larval development. This study focuses on basic attributes of developmental performance for larvae, but many other factors could be explored using this framework, including caste determination or even adult performance following development.

## MATERIALS AND METHODS

### Egg collection and transfer of larvae to *in vitro* rearing conditions

We collected *A. mellifera* eggs for *in vitro* rearing from a single hive maintained in an agricultural setting near Fargo, Cass County, North Dakota in the summer of 2014 (GPS 46°54′34.7″N 96°51′03.0″W). The study hive, along with three non-study hives, was placed in a wooded hedgerow near alfalfa and canola fields in the early summer. When the abundance of forage crops was low, hives were supplemented with pollen substitute (Mann Lake, MN, USA) and a 1:1 fructose-water solution (Brushy Mountain Bee Farm, NC, USA). Otherwise, adult bees were permitted to freely forage without supplementation. To collect eggs, the queen was captured in a 2 ml plastic vial and placed into a Jenter box (Blue Sky Bee Supply, Ravenna, OH, USA). The Jenter box was then mounted into the middle of the hive. After 24 h, the queen was released and the newly laid eggs were left for an additional 69 h until the box contained 0-21 h old larvae. The Jenter box was then collected into a polyethylene box and covered with water-saturated gauze to prevent desiccation.

Individual larvae were transplanted into the wells of a 48-well plate using a size 0 paintbrush. To transplant larvae from Jenter box to rearing plates, the paintbrush was placed under the dorsal end of each larva and lifted. Larvae were then placed carefully into well plates that had been provisioned with 10 µl of artificial food (see below for details of diet composition). The paintbrush was washed with distilled water before transplanting each larva and cleaned with 70% ethanol between each successive plate.

### Artificial diet treatments

To test the effects of larval nutrition on development, we provided honey bee larvae with one of nine artificial diets that varied in their relative macronutrient contents (Table 3). The macronutrient manipulation treatments centered on the artificial diet developed by Aupinel et al. (2005), which was used as the medium-medium treatment in this study. We chose this artificial diet and general rearing technique because it has been shown to result in development of worker bees (Aupinel et al., 2005; Kaftanoglu et al., 2011), which reduced the potential confounding factor of comparing individuals developing into different social castes (but see De Souza et al., 2015). Using the Aupinel et al. (2005) recipe as a starting point, protein content of the artificial diets was increased or decreased by incorporating more or less royal jelly (Pure Royal Jelly eBeeHoney.com, Ashland, OH, USA).

**Table 3.**
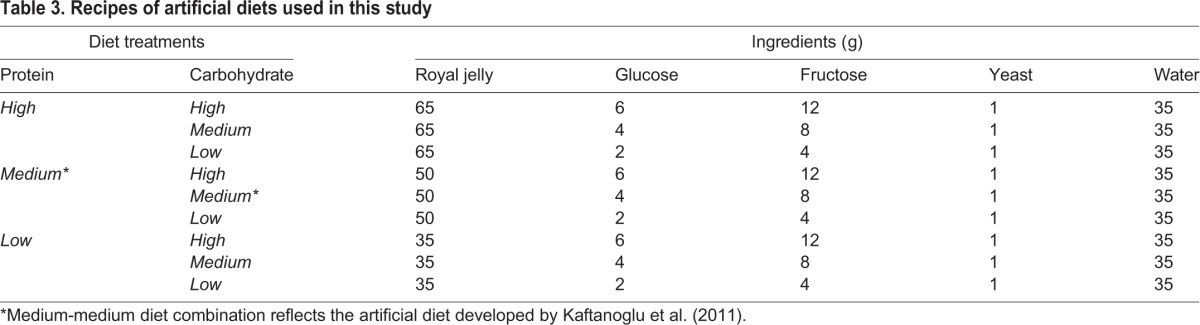
**Recipes of artificial diets used in this study**

Royal jelly contains many essential macronutrients and vitamins including carbohydrate and lipids important for larval nutrition (Wang et al., 2015). Royal jelly is the primary natural source of protein for developing *A. mellifera* larvae, so this study manipulated jelly content of the artificial food as a proxy for manipulating protein content. Carbohydrate content was simultaneously altered by increasing or decreasing the total amount of glucose and fructose added to jelly. A 1:2 ratio of glucose to fructose was used in all diet treatments (Table 3), but different absolute quantities were added to create high, medium, and low carbohydrate conditions. Protein and carbohydrate treatments were fully crossed, resulting in nine different diets (Table 3). For all diets in this study, we homogenized all ingredients for 10 min and stored at –80°C. Individual diet aliquots were thawed at room temperature prior to feeding.

### Determination of protein, carbohydrate, lipid, and water contents in artificial diets

Royal jelly is the primary source of protein, but it also contains some carbohydrates and water. We quantified the relative amounts of protein, carbohydrates and water in the commercial royal jelly used for the diets in order to account for the absolute quantity of each of these macronutrients across the nine diets. For example, the carbohydrate content of the ‘medium protein-medium carbohydrate’ diet includes the carbohydrate contribution from both the royal jelly and the added sugar.

Protein content of royal jelly was measured using a standard colorimetric Bradford assay (Sigma-Aldrich, MO, USA). Following protocols, a 200 mg/ml protein albumin solution was prepared and then diluted standards by dissolving 1 µl, 2.5 µl and 5 µl of the albumin solution in 1 ml of distilled H_2_O. We diluted 1 ml of royal jelly in 10 ml of water. We plated 5 µl aliquots of each of our three standards and diluted royal jelly into a 96-well plate. We added 250 µl of Bradford reagent to each well and left the colorimetric assay to develop for 20 min at room temperature. We read the absorbance of our samples at 595 nm using a spectrophotometer, and calculated royal jelly protein contents based on our determined standard absorbance curve.

Carbohydrate and water contents of the jelly were estimated using differential scanning calorimetry (DSC) (Sopade et al., 2004). One to five mg of the royal jelly sample was sealed in a Perkin-Elmer aluminum DSC pan. The aluminum pan was then placed into the differential scanning calorimeter (Perkin Elmer DSC Pyris 1, Waltham, MA, USA) along with an empty sealed pan that served as a control. During the scan, the sample chamber was perfused with helium gas at 10 ml/min. The samples were scanned between 25°C and −100°C at a rate of 1°C/min using a liquid-based cooling accessory (Perkin Elmer Cryofill™, Waltham, MA, USA) to determine both freezing and melting characteristics. Water melts near 0°C, generating an endothermic peak in the calorimeter scan, and the sugars caused distinct glass transitions between −20°C and −40°C. The area of the peaks generated during these events were calculated and used to determine the water content.

The freezing point depression was measured in royal jelly using a calibration based on glucose and fructose solutions in deionized water (5:7%, 7:10%, and 10:12% of glucose:fructose). In an Eppendorf tube, 100 µl of royal jelly was diluted in 900 µl of 100% ethanol, vortexed for 1 min, and chilled for 2 h at −80°C. Then the tubes were centrifuged at 14,000 rpm (18,800 ***g***) for 30 min. The supernatant was removed, dried in flowing nitrogen gas, and reconstituted in 1 ml of deionized water. Five microliters of this solution were analyzed in the DSC in quintuplicates, and the freezing and melting points were noted and compared against the above described glucose:fructose standards to obtain a gross estimate of the sugar content in royal jelly.

### Larval rearing on different diet treatments

Larvae (*n*=24 per diet) were reared for the entirety of larval growth in 48-well plates that were stored inside of modular incubator chambers (Billups-Rothenberg, del Mar, CA, USA), containing a small volume of 96% K_2_SO_4_ to maintain high humidity. Chambers were kept at a constant 34°C in a dark environmental chamber. Each day, larvae were removed from the environmental chamber and an additional day-specific volume of artificial diet was added to each well as follows: days 1 and 2, 10 µl; day 3, 20 µl; day 4, 30 µl; day 5, 40 µl; and day 6, 50 µl. Each larva was provisioned with a total of 160 µl of artificial diet. Larvae were kept in these conditions until they completely finished consuming their provisions, at which time they were removed from well plates and moved to pupation plates or declared dead and removed from the study. Death was indicated by repeated days of immobility, lack of growth, and blackened appearance.

### Data analysis and presentation of data

Statistical analyses were performed using R version 3.1.3 (R Core Team). Use of additional R packages are reported below where appropriate.

#### Survival

Larval survival was monitored daily until death or attainment of the prepupal stage. From this, we calculated the proportion of individuals that survived for each treatment and compared survival among different diet treatments using the pairwise proportion test. We analyzed the effects of protein and carbohydrate content (%) on survival using a generalized linear model with a ‘probit’ link function. The response variable was survival, scored as a binomial (1,0), and calculated protein and carbohydrate content (%) were the explanatory variables (see Table 1). As is standard for geometric analysis of nutrition (Le Gall and Behmer, 2014; Lee, 2007; Lee et al., 2008), this model included quadratic terms for protein and carbohydrate, as well as an interaction term between protein and carbohydrate. We then analyzed the effects of model terms on survival using analysis of deviance and likelihood ratio tests. Post hoc among-treatment group tests were performed using pairwise comparison of proportions.

#### Growth rate

At the time of daily feeding and maintenance, growth rates of six larvae from each artificial diet treatment were measured. The number of individuals assayed for growth rate was limited because extra handling increased mortality in preliminary studies. Each larva was photographed under a dissecting microscope with a mounted Moticam10 (Motic America, Richmond, BC, Canada). We measured axial body length (mm) using standard analysis tools in ImageJ (Rasband, U.S. National Institute of Health, Bethesda, MD, USA). We calculated the mean body length for each treatment so that growth trajectories could be visualized. Maximum larval length (*L_max_*) for each individual was determined regardless of whether they had lived or died throughout the assay. Each individual's relative growth rate (*G_R_*) was calculated as the difference between natural log-transformed *L_max_* and initial length (*L_0_*), divided by the time it took to reach *L_max_* (*T_max_*):

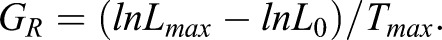
We modeled the effects of protein and carbohydrates on *G_R_* using a multiple linear regression model. Because mortality varied among different diet treatments, potentially confounding our results, each individual's survival score was included in our model to assess how growth rates varied between individuals that survived versus individuals that died. We then assessed differences in growth rate among different artificial diet treatments using analysis of variance, and post hoc comparisons were conducted using Tukey's test for honest significant differences (HSD).

#### Development time

For all surviving individuals in our study, we calculated development time (*T_D_*) as the time at which they attained the prepupal stage after consuming all provisioned larval resources. Preliminary data analysis showed that development times had a strong right-tailed distribution, and furthermore, sample sizes varied significantly among treatments due to differences in mortality. Because of these statistical considerations, *T_D_* was modeled as a response of protein and carbohydrate content using a generalized linear model with a Poisson link function because development time represents a count of days for larval growth. We included protein content (%), carbohydrate content (%), quadratic terms for both protein and carbohydrate contents, and an interaction between protein and carbohydrate content as explanatory variables in our model. We then assessed model terms using analysis of deviance and likelihood ratio tests.

#### Performance landscapes

To determine how honey bee larval development is affected by varying levels of macronutrients in the provisions, a ‘response-surface methodology’ was implemented (Le Gall and Behmer, 2014; Lee et al., 2008) to visualize how survival, growth rate, and development time change with varying quantities and ratios of protein and carbohydrates. Survival, growth rate, and development rates were mapped onto protein-carbohydrate performance landscapes (Le Gall and Behmer, 2014; Lee et al., 2008). This was achieved by fitting a thin plate spline surface to our data using the ‘fields’ package for R (Nychka et al., 2015).
